# A cross-sectional study of health-related quality of life deficits in individuals with comorbid diabetes and cancer

**DOI:** 10.1186/1477-7525-4-17

**Published:** 2006-03-22

**Authors:** Samantha L Bowker, Sheri L Pohar, Jeffrey A Johnson

**Affiliations:** 1 Department of Public Health Sciences, Faculty of Medicine and Dentistry, University of Alberta, 13-103 Clinical Sciences Building, Edmonton, Alberta, T6G 2G3, Canada; 2 Institute of Health Economics, #1200, 10405 Jasper Avenue, Edmonton, Alberta, T5J 3N4, Canada

## Abstract

**Background:**

Numerous studies have identified a reduced health related quality of life (HRQL) in patients that have either diabetes or cancer. We assessed the HRQL burden in patients with these comorbid conditions, postulating that they would have even greater HRQL deficits.

**Methods:**

Data from the Public Use File of the Canadian Community Health Survey (PUF CCHS) Cycle 1.1 (September 2000–November 2001) were used for this analysis. The total sample size of the CCHS PUF is 130,880 individuals. We used the Health Utilities Index Mark 3 (HUI3) to assess HRQL in patients with: 1) comorbid diabetes and cancer, 2) diabetes alone, 3) cancer alone, and 4) no diabetes or cancer. Analysis of covariance was used to compare the mean overall HUI3 score, controlling for age, sex, marital status, body mass index (BMI), physical activity level, smoking status, education level, depression status, and other chronic conditions.

**Results:**

We identified 113,587 individuals (87%) with complete data for the analysis. The comorbid diabetes and cancer group were older and a larger proportion reported being obese, inactive, having less than a secondary education and more chronic conditions when compared to the other three cohorts (p < 0.0001). However, the diabetes and cancer cohort was less likely to be depressed (p < 0.0001). Overall HUI3 scores were significantly lower for the diabetes and cancer group (unadjusted mean (SD): 0.67 (0.30)), compared to diabetes (0.78 (0.27)), cancer (0.78 (0.25)), and the reference group (0.89 (0.18)) (p < 0.0001). After adjusting for covariates, the comorbid diabetes and cancer group continued to have significantly lower overall HUI3 scores than the reference group (unstandardized mean difference: -0.11, 95% CI: -0.13 to -.0.09) (p < 0.0001).

**Conclusion:**

Individuals with diabetes and cancer had a clinically important and significantly lower HRQL than those with either condition alone. A better understanding of the relationship between diabetes and cancer, and their associated comorbidities, complications, and HRQL deficits may have important implications for prevention and management strategies.

## Background

Diabetes is a chronic medical condition that affects approximately 5% of Canadians aged 20 years and older, with type 2 diabetes accounting for 90% of all diagnosed cases of diabetes [1]. Type 2 diabetes is associated with several microvascular complications, such as retinopathy, nephropathy, and neuropathy, and macrovascular complications, such as heart disease, which results in significant morbidity and mortality [2-4]. A reduced self-reported health status or health-related quality of life (HRQL) reflects the significant health burden in this patient population [5-8].

In addition to the commonly recognized micro- and macrovascular complications, diabetes is associated with other comorbidities. A number of epidemiologic studies have identified an increased risk of developing cancer in people with type-2 diabetes [9-12]. The association appears to be mediated through the metabolic syndrome (also known as the insulin resistance syndrome). The metabolic syndrome is present in almost one-half of all older individuals and is a condition associated with hyperinsulinemia, insulin resistance and a predilection to type 2 diabetes [13].

It has been suggested that hyperinsulinemia combined with insulin resistance might promote carcinogenesis [14,15]. Several types of cancers have been found to be associated with type 2 diabetes, such as breast cancer [10,11], endometrial cancer [16,17], pancreatic cancer [11], and colorectal cancer [11,12].

The potential HRQL deficits associated with patients who have both type 2 diabetes and cancer may be quite large. There is extensive evidence in the literature of a reduced HRQL in patients with cancer [18,19]. However, despite the recognition of the link between type 2 diabetes and cancer, very little is known about HRQL in individuals with these comorbid chronic conditions. The objective of this study was to assess the HRQL burden in the following groups: 1) Individuals with comorbid diabetes and cancer compared to individuals with either condition alone, and 2) Individuals with comorbid diabetes and cancer compared to individuals without either condition. In both cases, we hypothesized that individuals with comorbid diabetes and cancer would have a significantly worse HRQL.

## Methods

### Canadian Community Health Survey (CCHS)

Data from the Public Use File of the Canadian Community Health Survey (CCHS PUF) Cycle 1.1 were used for this analysis. The CCHS contains information related to self-reported health determinants, health care utilization, and health status for the Canadian population. Data collection for the CCHS Cycle 1.1 occurred over a two-year, repeating cycle between September 2000 and November 2001 [20]. The CCHS targets individuals aged 12 years or older who are living in private residences in the ten provinces and the three territories. Persons living on Indian Reserves or Crown lands, residents of institutions, full-time members of the Canadian Armed Forces, exclusive cellular phone users, and residents of certain remote regions are excluded from this survey [20].

The CCHS uses a multistage stratified cluster design and a random digit dialing sampling method for selecting their sample [20]. The CCHS covers approximately 98% of the Canadian population aged 12 or older. Selection of individual respondents was designed to ensure over-representation of youths (12 to 19 years old) and seniors (65 years or older). Each respondent was assigned a weight to represent his or her contribution to the total population. The weights were used to derive estimates for all characteristics surveyed [20].

### Sample

**Figure 1:**
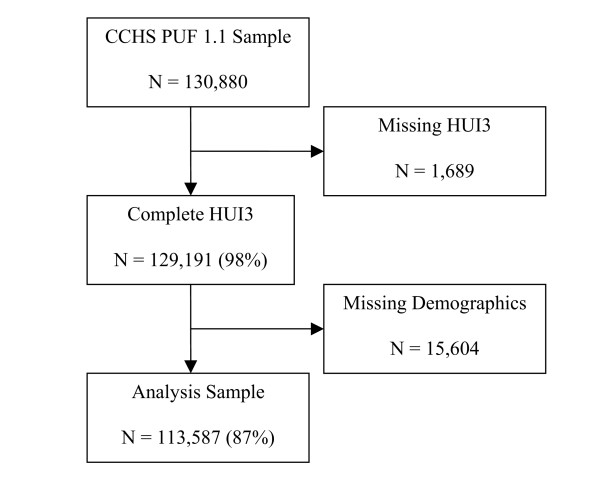
Survey Sample, Analysis Sample, and Missing Data.

The total sample size of the CCHS PUF is 130,880 individuals; 113,587 individuals (86.8%) had complete data for the analysis. Respondents were missing information for the following variables: HUI3 (N = 1,689; 1.3%), diabetes/cancer (N = 164; 0.1%), marital status (N = 162; 0.1%), BMI (N = 3,019; 2.3%), physical activity level (N = 8,461; 6.5%), smoking status (N = 139; 0.1%), education level (N = 1,264; 1.0%), depression status (N = 3,981; 3.0%), and number of chronic medical conditions (N = 1,185; 0.9%). Several respondents had missing information on more than one variable used in the analyses (Figure 1).

We then identified four groups of respondents, based on self-reported chronic disease status: 1) comorbid diabetes and cancer (unweighted N = 207; 0.2%), 2) diabetes alone (unweighted N = 4,394; 3.9%), 3) cancer alone (unweighted N = 1,692; 1.5%), and 4) a reference group without diabetes or cancer (unweighted N = 107,295; 94.5%).

### Health Utilities Index Mark 3 (HUI3)

The Health Utilities Index Mark 3 (HUI3) is an indirect preference-based measure of overall HRQL that is included in the CCHS PUF Cycle 1.1. The HUI has been used in hundreds of clinical studies covering a wide variety of health problems and in numerous large general population surveys since 1990 [7,21,22]. HUI measures have strong theoretical foundations, and are valid, reliable, and well accepted by patients and professionals [21,23,24]. The HUI3 is a useful measure for capturing HRQL in patients with comorbid diabetes and cancer. There is increasing evidence of the use of the HUI3 in individuals with type 2 diabetes [25-27]. There is also a substantial amount of research has used the HUI as an outcome measure for cancer [18,19,28,29]. However, there is no evidence in the literature of the use of the HUI in individuals with comorbid diabetes and cancer.

The HUI3 includes a comprehensive generic health status classification (i.e. profile) system and a utility scoring function [30,31]. The HUI3 administered for the CCHS Cycle 1.1 was a 31-item questionnaire. The classification system is comprised of 8 attributes: vision, hearing, speech, ambulation, dexterity, emotion, cognition, and pain. Each attribute has 5 or 6 levels of functioning, thereby defining 972,000 possible unique health states [21]. It is important to note that the CCHS PUF has suppressed the single-attribute utility scores, thus precluding evaluation of the impact of diabetes and cancer on any single attributes in this analysis. The overall HUI3 scoring system provide utility (preference) scores on a generic scale ranging from -0.36 to 1.00, where worst possible health = -0.36, dead = 0.00, and perfect health = 1.00 [21].

Differences of 0.03 or greater in the mean overall HUI3 scores are considered clinically important [21]. Other studies have confirmed this value as clinically important [27,32,33]. The basis for this clinically important difference of 0.03 or greater is that a change in one level of functioning on any of the eight attributes is considered to be qualitatively important [34]. Therefore, 0.03 represents the smallest difference in the overall score resulting from a one level change in functioning on one attribute (e.g. the difference in overall score between having a Level 1 and Level 2 functioning on the vision attribute) [8].

### Statistical analyses

Descriptive statistics were used to compare our study groups; comparisons were evaluated using ANOVA for continuous variables and chi-square tests for categorical variables. ANCOVA was used to compare the mean overall HUI3 score in each of the four cohorts while controlling for potential confounders. The following covariates were adjusted for in the model: age, sex, marital status, body mass index (BMI), physical activity level, smoking status, education level, depression status (from the Composite International Diagnostic Interview Short Form for Major Depression (CIDI)), and number of chronic medical conditions other than diabetes or cancer. Income level was not used as a covariate because there was too much missing data (>10% of the population had missing data on this variable). All data were from self-report.

Age was categorized into quartiles (12–29 years, 30–44 years, 45–59 years, and ≥60 years). For marital status, individuals were categorized as "married/common-law" or "widowed/separated/divorced/single". Respondents' BMI was categorized as not obese (BMI < 30) or obese (BMI ≥ 30) [35]. Physical activity level was categorized as "active", "moderately active", or "inactive". Smoking status was categorized as "daily", "occasionally", or "not at all". Education level was categorized as "less than secondary school graduation", "secondary school graduate", "other post-secondary school" (e.g. diploma/certificate from a trade school, some community college), or "post-secondary school graduate" (e.g. college or university degree).

Respondents were categorized as "depressed" or "not depressed" according to the Composite International Diagnostic Interview (CIDI) Short Form for Major Depression. Respondents who had a predicted probability of 0.90 or greater for major depression on the CIDI were considered to have depression [36]. This is in accordance with the DSM-IV diagnostic criteria for a major depressive disorder [36]. Depression is an important comorbidity to include in the analyses, as it has been linked to a reduced HRQL in patients with diabetes and in patients with cancer [37,38]. Lastly, number of chronic medical conditions other than diabetes or cancer was categorized as follows: 0, 1, 2, or ≥3 chronic conditions. For all comparisons, a p-value of less than 0.05 (two-tailed) was considered to be statistically significant. Normalized sampling weights were used in the analysis to account for unequal selection probability. All statistical analyses were performed using SPSS Version 13.0.

## Results

In our sample of 113,587 individuals, all of the patient characteristics were significantly different for the four groups (Table 1). In general, the comorbid diabetes and cancer group tended to be older (70.4% of respondents were ≥60 years old) compared to the others. There were significantly fewer men in the cancer alone group (43.7%) compared to the other groups, where men represented approximately 50% of the population. Approximately one-third of respondents were obese (BMI ≥ 30) in the comorbid diabetes and cancer and the diabetes groups. A significantly larger proportion of patients reported being inactive and having less than a secondary education in the comorbid diabetes and cancer and the diabetes groups, compared to the cancer alone or the reference group. Interestingly, the reference group responded that they smoked more frequently than the others (approximately 27% of respondents were daily or occasional smokers). The cancer group had a significantly larger proportion of individuals who were considered to be depressed (12.5%). Lastly, the diabetes and cancer group self-reported more chronic conditions than the other groups.

**Table 1:** Patient Characteristics Stratified by Disease Group (N = 113,587)

	Diabetes and Cancer (*N = 207))	Diabetes (*N = 4,394)	Cancer (*N = 1,692)	No Diabetes or Cancer (*N = 107,295)
HUI3 (Overall Score)**				
Mean (Standard Deviation, SD)	0.67 (0.30)	0.78 (0.27)	0.78 (0.25)	0.89 (0.18)
Median (Range)	0.78 (-0.24 – 1.0)	0.91 (-0.31 – 1.0)	0.91 (-0.21 – 1.0)	0.97 (-0.31 – 1.0)
Age (Years), (%)^†^				
12–29 Years	2 (1.0%)	144 (3.3%)	81 (4.8%)	32,018 (29.8)
30–44 Years	4 (1.9%)	554 (12.6%)	189 (11.2%)	32,708 (30.5%)
45–59 Years	55 (26.7%)	1,325 (30.2%)	446 (26.4%)	24,933 (23.2%)
≥60 Years	145 (70.4%)	2,370 (53.9%)	976 (57.7%)	17,636 (16.4%)
Men, (%)^†^	114 (55.3%)	2,256 (51.3%)	740 (43.7%)	51,726 (48.2%)
BMI, (%)^†^				
<30	139 (67.1%)	2,873 (65.4%)	1,442 (85.2%)	93,511 (87.2%)
≥30	68 (32.9%)	1,520 (34.6%)	250 (14.8%)	13,784 (12.8%)
Marital Status, (%)^†^				
Married/Common Law, (%)	147 (71.4%)	2,933 (66.8%)	1,143 (67.6%)	61,595 (57.4%)
Widowed/Separated/Divorced/Single	59 (28.6%)	1,461 (33.2%)	549 (32.4%)	45,700 (42.6%)
Physical Activity Level, (%)^†^				
Active	29 (14.0%)	663 (15.1%)	330 (19.5%)	25,257 (23.5%)
Moderately Active	26 (12.6%)	937 (21.3%)	368 (21.8%)	25,542 (23.8%)
Inactive	152 (73.4%)	2,794 (63.6%)	993 (58.7%)	56,496 (52.7%)
Education Level, (%)^†^				
<Than Secondary	95 (45.9%)	1,806 (41.1%)	544 (32.2%)	29,837 (27.8%)
Secondary Graduate	21 (10.1%)	743 (16.9%)	313 (18.5%)	20,204 (18.8%)
Other Post-Secondary	14 (6.8%)	270 (6.1%)	95 (5.6%)	9,151 (8.5%)
Post-Secondary Graduate	77 (37.2%)	1,574 (35.8%)	740 (43.7%)	48,102 (44.8%)
Smoking Status, (%)^†^				
Daily	38 (18.4%)	734 (16.7%)	271 (16.0%)	23,527 (21.9%)
Occasionally	3 (1.4%)	117 (2.7%)	51 (3.0%)	5,066 (4.7%)
Not At All	166 (80.2%)	3,542 (80.6%)	1,369 (81.0%)	78,703 (73.4%)
Depressed, (%)^†^	10 (4.8%)	342 (7.8%)	212 (12.5%)	7,837 (7.3%)
Number of Chronic Conditions, (%)^†^				
0	8 (3.9%)	675 (15.4%)	305 (18.0%)	40,775 (38.0%)
1	26 (12.6%)	1,002 (22.8%)	360 (21.3%)	29,471 (27.5%)
2	30 (14.6%)	864 (19.7%)	314 (18.6%)	17,977 (16.8%)
≥3	142 (68.9%)	1,853 (42.2%)	712 (42.1%)	19,072 (17.8%)

*Unweighted N (All other values in the table are weighted).

**P < 0.0001 for Analysis of Variance (ANOVA)

^†^P < 0.0001 for Chi-square test

The unadjusted mean (standard deviation, SD) overall HUI3 for the comorbid diabetes and cancer group was 0.67 (0.30). This value was significantly lower than that for the other groups: 0.78 (0.27) for diabetes alone, 0.78 (0.25) for cancer alone, and 0.89 (0.18) for the reference group (Table 1).

After adjusting for the covariates, the comorbid diabetes and cancer group had a significantly lower overall HUI3 score compared to the reference group, which served as the reference group (mean difference: -0.11, 95% CI: -0.13 to -0.09; Table 2). As hypothesized, the comorbid diabetes and cancer group also had a significantly lower HUI3 than the diabetes (mean difference: -0.04, 95% CI: -0.05 to -0.04) and the cancer (mean difference: -0.04, 95% CI: -0.05 to -0.03) groups. All of these between group differences would be considered clinically important.

**Table 2:** Weighted ANCOVA for HUI3 by Disease Group

	B* (Unstandardized Mean Difference)	95% Confidence Interval (Lower – Upper)	
Diabetes/Cancer**			
Diabetes and Cancer	-0.108^†^	-0.131	-0.086
Diabetes	-0.040^†^	-0.045	-0.035
Cancer	-0.040^†^	-0.048	-0.033
No Diabetes Or Cancer	reference	-	-
Age**			
12–29 Years	0.056^†^	0.052	0.059
30–44 Years	0.038^†^	0.035	0.041
45–59 Years	0.021	0.018	0.024
≥60 Years	reference	-	-
Sex**			
Male	-0.011	-0.013	-0.009
Female	reference	-	-
Marital Status**			
Married/Common Law	0.023	0.021	0.025
Widow/Separated/Divorced/Single	reference	-	-
Physical Activity Level**			
Active	0.038^†^	0.036	0.041
Moderately Active	0.032^†^	0.029	0.034
Inactive	reference	-	-
Education Level**			
<Than Secondary Education	-0.040^†^	-0.043	-0.038
Secondary Education Graduate	-0.011	-0.014	-0.009
Other Post-Secondary Education	-0.011	-0.015	-0.007
Post-Secondary Education Graduate	reference	-	-
Smoking Status**			
Daily Smoker	-0.020	-0.023	-0.018
Occasional Smoker	-0.011	-0.016	-0.007
Not At All	reference	-	-
BMI**			
<30	0.015	0.012	0.018
≥30	reference	-	-
Depression Status**			
Not Depressed	0.142^†^	0.138	0.145
Depressed	reference	-	-
Number of Chronic Conditions**			
0	0.149^†^	0.146	0.152
1	0.127^†^	0.124	0.130
2	0.094^†^	0.091	0.097
≥3	reference	-	-

*Adjusted for all other covariates in the table

**All variables were statistically significant, P < 0.0001

^†^Clinically meaningful difference of ≥0.03

Respondents who were younger, married/common law, physically active, not obese, not depressed, and had no chronic medical conditions had significantly higher overall HUI3 scores (Table 2). On the other hand, males, smokers, or respondents that had not completed high school had significantly lower overall HUI3 scores (Table 2). Of note, there were clinically important differences in the following variables for overall HUI3 score: age, physical activity level, education level, depression status, and number of chronic medical conditions (Table 2).

## Discussion

We used population-based data from the Canadian Community Health Survey Cycle 1.1 to determine HRQL, using the overall HUI3 score, in respondents with self-reported diabetes and cancer compared to a reference group without diabetes or cancer. As hypothesized, we found that patients with diabetes and cancer had a significantly lower and clinically important difference in overall HUI3 score compared to respondents who had no diabetes or cancer, even after controlling for potential confounding variables.

All covariates included in the model were associated with significant differences in overall HRQL; however, not all of the variables revealed clinically important differences. Number of chronic medical conditions and specific comorbidities, such as depression, had the largest impact on HRQL. Respondents who were not depressed had a 0.14 higher HUI3 overall score compared to respondents who were depressed. Also, the fewer chronic medical conditions apart from diabetes and cancer that respondents had, the higher their overall HUI3 score (0.15 for no chronic medical conditions compared to respondents who had 3 or more chronic medical conditions). These findings are in agreement with Maddigan et al, who found that comorbidities such as cardiovascular disease and depression had the largest impact on HRQL in patients with type 2 diabetes [7,8].

Previous research has reported lower overall HUI3 scores in individuals that have either type 2 diabetes [7,25] or cancer [18,19]. These deficits are likely a result of the complications, comorbidity, and treatment regimens associated with these chronic conditions. There is strong evidence of an association between type 2 diabetes and cancer; this association appears to be mediated through the metabolic syndrome [9-11]. Furthermore, there is an increased mortality for patients with comorbid diabetes and cancer [9,39]. Despite these associations, however, very little is known about the HRQL deficits in this patient population.

The overall HUI3 scores we observed in our sample are similar to that of other studies. A study by Maddigan et al, observed the same mean overall HUI3 score of 0.78 in patients with type 2 diabetes as our study did in patients that have diabetes; this finding is not surprising considering the authors also used CCHS data [32]. They also observed mean overall HUI3 scores of 0.77 for arthritis patients (a figure similar to the 0.78 we found for our cancer group), 0.54 for patients who have had a stroke, and 0.90 for the general population (a figure similar to the 0.89 we found for our reference group that did not have diabetes or cancer) [32].

There are some limitations inherent in this study. Firstly, the cross-sectional study design of the CCHS is not ideal for the purpose of answering this question. A longitudinal study design would more appropriately address this question, as changes in HRQL over time could also be assessed. Since both are chronic conditions, however, the duration of follow-up would be prohibitive. Furthermore, as a cross-sectional study, in conditions which have substantial mortality such as cancer, there is likely a survival bias and respondent bias, with only those more healthy individuals in the community being respondents. This would, of course, lead to an over estimate of the respondents in the cancer groups.

We also lacked information on potentially useful clinical variables. Recent research has revealed that waist circumference or waist to hip ratio may be more effective than BMI in predicting risk for type 2 diabetes or cancer [40,41]. Furthermore, there is evidence that obesity, as measured by waist to hip ratio and/or waist circumference, are associated with various measures of HRQL [42,43]. It would also be useful to have clinical information on the different types of cancer and on disease severity for both diabetes and cancer, all of which would differentially impact the HRQL of individuals [26]. Although type of cancer and a variable that allows calculation of time since cancer diagnosis are collected as part of the CCHS Cycle 1.1 microdata, these variables are not available in the CCHS PUF.

Another limitation of this dataset was the inability to separate the diabetes cohort into type 1 diabetes and type 2 diabetes. The group with type 2 diabetes may have been of particular interest, as there is well-documented evidence of an increased risk of various types of cancer in patients with type 2 diabetes [10-12,17]. However, there is no literature to support the evidence of a link between type 1 diabetes and cancer, and we could expect that approximately 10% of the diabetes cohort has type 1 diabetes [1,8]. Insulin use is available in the CCHS PUF, there is a substantial amount of missing data on this variable (95.2% or 124,609/130,880). Also, because the variables in the CCHS are self-reported, there is a potential for recall bias or social desirability bias. For example, there is evidence that respondents may respond in a socially desirable manner when self-reporting their BMI (height and weight) [44], smoking status [45], and physical activity status [46].

We recognize that the PUF version of the CCHS data is not as precise as the micro data available through Statistics Canada. In the PUF, some data elements are aggregated, and variables that have been collapsed, and more importantly there is no information on the single attribute utility scores from the HUI3. There were also missing data on a number of the covariates (particularly for physical activity level, 6.5%; depression status, 3.0%; and BMI 2.3%). We noted that respondents with missing data on any of these three variables were younger and had lower overall HUI3 scores. There was no relationship between missing BMI and reporting diabetes or cancer. Respondents with cancer were more likely to be missing data on physical activity, although the differences were small. Respondents with both diabetes and cancer were more likely to be missing data on depression. As such, excluding these individuals likely overestimated the HUI3 scores across all groups. Of note, the diabetes and cancer group was most likely to have had missing data on depression; had those individuals been included, the overall HUI3 score would have been even lower. One variable in particular that had a substantial amount of missing data (>10% for all respondents) was income; therefore, this variable was not included in our analyses. It has been shown that income, as a determinant of health, is a distinct aspect of socioeconomic status that is useful in predicting HRQL, independent of education level [47].

Despite the above mentioned limitations in this study, there are some key strengths that must be recognized. Most importantly, the CCHS has a very large sample that is considered to be representative of 98% of the Canadian population. Also, we used the HUI3 as our measure of HRQL. There is evidence of the validity of this measure in people who have type 2 diabetes [27] and in people who have cancer [48]. It has also been used in other large national population health surveys [7,21,22]. Finally, to our knowledge, this is the first study that has assessed HRQL, using the HUI3 as an outcome measure, in patients who have comorbid diabetes and cancer.

## Conclusion

We found that patients with comorbid diabetes and cancer had a clinically meaningfully worse overall HUI3 score compared to respondents who had no diabetes or cancer, differences which were considerable, even after controlling for potential confounding variables. While our results are intriguing, they should be considered hypothesis-generating given the limitations inherent in this study. Diabetes (especially type 2 diabetes) and cancer are largely preventable through lifestyle. Separately, the public health burden of these two chronic diseases is large; together their burden is even greater. A better understanding of the relationship between diabetes and cancer, and their associated comorbidities and complications may have important implications for prevention and management of these chronic conditions. These prevention and management strategies will in turn have positive effects on the HRQL of individuals affected by these conditions.

## Competing interests

The author(s) declare that they have no competing interests.

## Authors' contributions

SLB carried out the analysis and interpretation of data, drafted and revised the manuscript. SLP interpreted the data and revised the manuscript. JAJ interpreted the data and revised the manuscript. All authors read and approved the final manuscript.

## Appendix 1: Identification of Chronic Conditions in CCHS PUF

The variable cccagtot lists the number of chronic conditions and is based on the variables ccca_011 to cccag221

**Table 3:** 

CCCA_011	(We are interested in long-term conditions that have lasted or are expected to last 6 months or more and that have been diagnosed by a health professional). Do you have food allergies?
CCCA_021	Do you have any other allergies?
CCCA_031	Do you have asthma?
CCCA_041	(Remember, we're interested in conditions diagnosed by a health professional). Do you have fibromyalgia?
CCCA_051	Do you have arthritis or rheumatism excluding fibromyalgia?
CCCA_061	(Remember, we're interested in conditions diagnosed by a health professional). Do you have back problems, excluding fibromyalgia and arthritis diagnosed by a health professional?
CCCA_071	Do you have high blood pressure?
CCCA_081	Remember, we're interested in conditions diagnosed by a health professional. Do you have migraine headaches?
CCCA_91A	(Remember, we're interested in conditions diagnosed by a health professional). Do you have chronic bronchitis?
CCCA_91B	Do you have emphysema or chronic obstructive pulmonary disease (COPD)?
CCCA_101	Do you have diabetes?
CCCA_111	Do you have epilepsy?
CCCA_121	Do you have heart disease diagnosed by a health professional?
CCCA_131	Do you have cancer?
CCCA_141	(Remember, we're interested in conditions diagnosed by a health professional). Do you have stomach or intestinal ulcers?
CCCA_151	Do you suffer from the effects of a stroke?
CCCA_161	Do you suffer from urinary incontinence?
CCCA_171	Do you have a bowel disorder such as Crohn's Disease or colitis?
CCCA_191	Do you have cataracts?
CCCA_201	Do you have glaucoma?
CCCA_211	Do you have a thyroid condition?
CCCA_251	Remember, we're interested in conditions diagnosed by a health professional. Do you have chronic fatigue syndrome?
CCCA_261	Do you suffer from multiple chemical sensitivities?
CCCAG221	Has other chronic condition

## References

[B1] Health Canada (2003). Responding to the challenge of diabetes in Canada. First report of the National Diabetes Surveillance System (NDSS). Ottawa, Health Canada.

[B2] The Diabetes Control and Complications Trial Research Group (1993). The effect of intensive treatment of diabetes on the development and progression of long-term complications in insulin-dependent diabetes mellitus. New Engl J Med.

[B3] Ahroni JH, Boyko EJ, Davignon DR, Pecoraro RE (1994). The health and functional status of veterans with diabetes. Diabetes Care.

[B4] U.K. Prospective Diabetes Study Group (1998). UKPDS 33: Intensive blood glucose control with sulphonylureas or insulin compared with conventional treatment and risk of complications in patients with type 2 diabetes. Lancet.

[B5] Hirsch A, Bartholomae C, Volmer T (2000). Dimensions of quality of life in people with non-insulin dependent diabetes. Qual Life Res.

[B6] Woodcock AJ, Julious SA, Kinmonth AL, Campbell MJ, Diabetes Care From Diagnosis Group (2001). Problems with the performance of the SF-36 among people with type 2 diabetes in general practice. Qual Life Res.

[B7] Maddigan SL, Feeny DH, Johnson JA (2005). Health-related quality of life deficits associated with diabetes and comorbidities in a Canadian National Population Health Survey. Qual Life Res.

[B8] Maddigan SL, Feeny DH, Majumdar SR, Farris KKB, Johnson JA Understanding Determinants of Health in Type 2 Diabetes. Institute of Health Economics Working Paper.

[B9] Saydah SH, Loria CM, Eberhardt MS, Brancati FL (2003). Abnormal glucose tolerance and the risk of cancer death in the United States. Am J Epidemiol.

[B10] Michels KB, Solomon CG, Hu FB, Rosner BA, Hankinson SE, Colditz GA, Manson JE, Nurses' Health Study (2003). Type 2 diabetes and subsequent incidence of breast cancer in the Nurses' Health Study. Diabetes Care.

[B11] Coughlin SS, Calle EE, Teras LR, Petrelli J, Thun MJ (2004). Diabetes mellitus as a predictor of cancer mortality in a large cohort of US adults. Am J Epidemiol.

[B12] Hu FB, Manson JE, Liu S, Hunter D, Colditz GA, Michels KB, Speizer FE, Giovannucci E (1999). Prospective study of adult onset diabetes mellitus (type 2) and risk of colorectal cancer in women. J Natl Cancer Inst.

[B13] Alexander CM (2003). The coming of age of the metabolic syndrome. Diabetes Care.

[B14] Rosenfeld RG (2003). Insulin-like growth factors and the basis of growth. N Engl J Med.

[B15] Kim YI (1998). Diet, lifestyle, and colorectal cancer: Is hyperinsulinemia the missing link?. Nutrition Reviews.

[B16] Weiderpass E, Persson I, Adami HO, Magnusson C, Lindgren A, Baron JA (2000). Body size in different periods of life, diabetes mellitus, hypertension, and risk of postmenopausal endometrial cancer (Sweden). Cancer Causes Control.

[B17] Maatela J, Aromaa A, Salmi T, Pohja M, Vuento M, Gronrros M (1994). The risk of endometrial cancer in diabetic and hypertensive patients: A nationwide record-linkage study in Finland. Ann Chir Gynaecol Suppl.

[B18] Barr RD, Pai M, Weitzman S, Feeny DH, Furlong WJ, Rosenbaum P (1994). A Multi-Attribute Approach to Health Status Measurement and Clinical Management – Illustrated by an Application to Brain Tumours in Childhood. Int J Oncology.

[B19] Barr RD, Feeny DH, Furlong WJ, Weitzman S, Torrance GW (1995). A Preference-Based Approach to Health-Related Quality of Life in Children with Cancer. Int J Pediatric Hematology/Oncology.

[B20] Beland Y (2002). Community Health Survey – Methodological Overview. Health Reports.

[B21] Horsman J, Furlong W, Feeny D, Torrance G (2003). The Health Utilities Index (HUI): concepts, measurement properties and applications. Health Qual Life Outcomes.

[B22] Rizzo JA, Pashko S, Friedkin R, Mullahy J, Sindelar JL (1998). Linking the Health Utilities Index to National Medical Expenditure Survey Data. PharmacoEconomics.

[B23] Gemke RJBJ, Gouke JB (1996). Reliability and Validity of a Comprehensive Health Status Measure in a Heterogeneous Population of Children Admitted to Intensive Care. J Clin Epidemiology.

[B24] Jones CA, Feeny D, Eng K (2005). Test-Retest Reliability of Health Utilities Index Scores: Evidence from Hip Fracture. Int J Technol Assess Health Care.

[B25] Maddigan SL, Feeny DH, Johnson JA (2003). A Comparison of the Health Utilities Index Mark 2 and Mark 3 in Type 2 Diabetes. Med Decis Making.

[B26] Maddigan SL, Majumdar SR, Toth EL, Feeny DH, Johnson JA, the DOVE Investigators (2003). Health related quality of life deficits associated with varying degrees of disease severity in type 2 diabetes. Health Qual Life Outcomes.

[B27] Maddigan SL, Feeny DH, Johnson JA (2004). Construct Validity of the RAND-12 and Health Utilities Index Mark 2 and Mark 3 in Type 2 Diabetes. Qual Life Res.

[B28] Feeny DH, Furlong WJ, Barr RD, Torrance GW, Rosenbaum P, Weitzman S (1992). A Comprehensive Multi-Attribute System for Classifying the Health Status of Survivors of Childhood Cancer. J Clin Oncology.

[B29] Feeny DH, Leiper A, Barr RD, Furlong WJ, Torrance GW, Rosenbaum P, Weitzman S (1993). The Comprehensive Assessment of Health Status in Survivors of Childhood Cancer: Application to High-Risk Acute Lymphoblastic Leukaemia. Br J Cancer.

[B30] Feeny DH, Furlong WJ, Torrance GW, Goldsmith CH, Zhu Z, DePauw S, Denton M, Boyle M (2002). Multi-Attribute and Single-Attribute Utility Functions for the Health Utilities Index Mark 3 System. Med Care.

[B31] Furlong WJ, Feeny DH, Torrance GW, Barr RD (2001). The Health Utilities Index (HUI) System for Assessing Health-Related Quality of Life in Clinical Studies. Ann Med.

[B32] Grootendorst P, Feeny D, Furlong W (2000). Health Utilities Index Mark 3 Evidence of Construct Validity for Stroke and Arthritis in a Population Health Survey. Med Care.

[B33] Drummond M (2001). Introducing Economic and Quality of Life Measurements into Clinical Studies. Ann Medicine.

[B34] Maddigan SL, Feeny DH, Majumdar SR, Farris KB, Johnson JA Understanding the Determinants of Health in Type 2 Diabetes. Am J Public Health.

[B35] Health Canada. Health risk classification according to body mass index (BMI). http://www.hc-sc.gc.ca/fn-an/nutrition/weights-poids/guide-ld-adult/bmi_chart_java-graph_imc_java_e.html.

[B36] Patten SB, Brandon-Christie J, Devji J, Sedmak B (2000). Performance of the Composite International Diagnostic Interview Short Form for Major Depression in a Community Sample. Chronic Dis Can.

[B37] Goldney RD, Phillips PJ, Fisher LJ, Wilson DH (2004). Diabetes, depression, and quality of life: a population study. Diabetes Care.

[B38] Ell K, Sanchez K, Vourlekis B, Lee PJ, Dwight-Johnson M, Lagomasino I, Muderspach L, Russell C (2005). Depression, correlates of depression, and receipt of depression care among low-income women with breast or gynaecologic cancer. J Clin Oncol.

[B39] Bowker SL, Majumdar SR, Veugelers P, Johnson JA (2006). Increased Cancer-Related Mortality for Patients With Type 2 Diabetes Who Use Sulfonylureas or Insulin. Diabetes Care.

[B40] Angleman SB, Harris TB, Melzer D (2006). The role of waist circumference in predicting disability in pre-retirement age adults. Int J Obes.

[B41] Macinnis RJ, English DR, Hopper JL, Gertig DM, Haydon AM, Giles GG (2006). Body size and composition and colon cancer risk in women. Int J Cancer.

[B42] Lopez-Garcia E, Banegas JR, Gutierrez-Fisac JL, Perez-Regadera AG, Ganan LD, Rodriguez-Artalejo F (2003). Relation between body weight and health-related quality of life among the elderly in Spain. Int J Obes Relat Metab Disord.

[B43] Bannerman E, Miller MD, Daniels LA, Cobiac L, Giles LC, Whitehead C, Andrews GR, Crotty M (2002). Anthropometric indices predict physical function and mobility in older Australians: the Australian Longitudinal Study of Ageing. Public Health Nutr.

[B44] Gillum RF, Sempos CT (2005). Ethnic variation in validity of classification of overweight and obesity using self-reported weight and height in American women and men: the Third Nation Health and Nutrition Examination Survey. Nutr J.

[B45] Lewis SJ, Cherry NM, McL Niven R, Barber PV, Wilde K, Povey AC (2003). Cotinine levels and self-reported smoking status in patients attending a bronchoscopy clinic. Biomarkers.

[B46] Motl RW, McAuley E, DiStefano C (2005). Is social desirability associated with self-reported physical activity?. Prev Med.

[B47] Dapeuto JJ, Servente L, Francolino C, Hahn EA (2005). Determinants of quality of life in patients with cancer. Cancer.

[B48] Glaser AW, Furlong W, Walker DA, Fielding K, Davies K, Feeny DH, Barr RD (1999). Applicability of the Health Utilities Index to a population of childhood survivors of central nervous system tumours in the UK. Eur J Cancer.

